# A Novel Leadership Curriculum for Emergency Medicine Residents

**DOI:** 10.21980/J81D2S

**Published:** 2024-01-31

**Authors:** Michael J Zdradzinski, Stephen Sanders, Qasim Kazmi, Vanessa Fields, James O’Shea, Sar Medoff

**Affiliations:** * Emory University School of Medicine, Department of Emergency Medicine, Atlanta, GA

## Abstract

**Audience and Type of Curriculum:**

This longitudinal leadership curriculum is designed for emergency medicine residents at all levels, with individual sessions designed for each residency year.

**Length of Curriculum:**

This curriculum runs once annually over three to four years of emergency medicine residency.

**Introduction:**

Leadership is a vital skill for emergency physicians but is often passively taught during residency training. Strong leadership skills can lead to improved patient outcomes, but very few residency programs in any specialty and no emergency medicine residency programs have published comprehensive leadership training curricula.

**Educational Goals:**

The goals of this curriculum are to expose Emergency Medicine residents to the basics of leadership, to provide a graduated series of interactive, psychologically safe environments to explore individual leadership styles, to review interesting relevant literature, and to discuss leadership principles and experiences with senior leaders in our Emergency Department.

**Educational Methods:**

The educational strategies used in this curriculum include: brief lecture-style seminars, small group discussion and reflection, and a panel-style discussion.

**Research Methods:**

The educational content of this curriculum was evaluated by learners via feedback surveys after each session.

**Results:**

Course evaluations conducted in both 2017 and 2020 showed that more than 89% of resident participants found these sessions “useful” or “very useful.” All residents surveyed agreed that leadership is an important topic for emergency medicine residency, and 76% felt that the inclusion of leadership content strengthened the residency’s curriculum. Suggestions for future topics included handling personal conflict and discussing transitions in leadership during yearly residency promotions.

**Discussion:**

The curriculum has been successfully implemented for seven years. It has proven to be sustainable and requires minimal resources. The residents report high satisfaction with the curriculum and agree that formal instruction on the topic of leadership is important to their on-shift performance and careers.

**Topics:**

Leadership, communication.

## USER GUIDE

[Table t1-jetem-9-1-c1]

**Table t1-jetem-9-1-c1:** 

List of Resources:	
Abstract	1
User Guide	3
Curriculum Chart	6
Appendix A: Curriculum Overview and Discussion Points	9
Appendix B: PGY1 Session Slideshow	14
Appendix C: PGY2 Session Slideshow	15

**Learner Audience:**

Interns, Junior Residents, Senior Residents

**Length of Curriculum:**

This longitudinal curriculum spans a 3-year period, beginning in spring of postgraduate year 1 (PGY-1) year and ending in spring of PGY-3 (or, optionally, PGY-4).

**Topics:**

Leadership, communication.

**Objectives:**

By the end of this curriculum, learners will be able to:

1. Understand the importance of deliberate leadership practice to success as an emergency physician.
2. Explain common leadership styles, their utility in certain clinical situations, and their relative benefits and drawbacks.
3. Understand specific behaviors and actions that can enhance or detract from a clinician’s leadership in the emergency department.

### Brief introduction

By the end of emergency medicine (EM) residency, trainees are expected to be competent clinicians, efficient administrators, and team leaders. Curricula are designed primarily toward diagnosis and treatment of disease, and very few residency programs explicitly train residents to be effective leaders.^1^ As a result, many physicians consider themselves “accidental leaders” who have honed their skills via direct observation of attending physicians or modeled behavior.^2^,^3^ Due to this lack of leadership education in physician training, residents are left to adopt habits and styles from those they observe rather than intentionally developing their own leadership styles.

Leadership skills play an outsize role in the Emergency Department. The presence of a large, dynamic clinical team, unpredictable patient volumes, and a diverse case mix means that an effective emergency physician must be a leader while on-shift. They must orchestrate the department’s ebbs and flows, prioritize tasks for the team to perform, and navigate difficult interpersonal situations. Despite the importance of leadership to physicians’ clinical success, it is seldom given time in packed residency conference curricula; instead, it tends to exist as part of the “hidden curriculum” of residency training. To our knowledge, no EM residency program currently has a general leadership training curriculum.

### Problem identification, general and targeted needs assessment

In 2015, the Accreditation Council for Graduate Medical Education (ACGME) published an article calling for a “Leadership Curriculum for Residents” with a “sequenced, asynchronous, online curriculum” paired with mentor and peer-group discussions, leadership capstone courses, and leadership workshops at annual education conferences.^2^ Models for leadership courses can be found in the military, Harvard Business School’s Authentic Leadership Development course, and the Center for Creative Leadership, among others, but very few postgraduate training programs have developed their own curricula. A few residency programs in specialties such as Internal Medicine and Otolaryngology have implemented resident leadership curricula based on the Harvard Business School and US Army philosophies.^4^,^5^ Prior to the initiation of this curriculum, there was no formal instruction regarding on-shift leadership skills within our department.

With no identified best-practices for teaching general leadership skills to EM residents, we utilized leadership resources from other sources to create a curriculum. Prior research using focus groups of medical professionals determined four important themes that characterize high-quality physician leadership: team management, vision, communication skills, and personal attributes.^6^ Building on these themes, we also incorporated aspects of Authentic Leadership, a leadership theory developed by Bill George, professor at Harvard Business School. This theory consists of a three part process of experience, reflection, and feedback.^7^ Additionally, we drew significant inspiration from a leadership course designed for second year internal medicine residents at Massachusetts General Hospital that includes modules on clinical leadership, leadership styles, Authentic Leadership, and effective team leadership.^4^ The EM literature includes one curriculum from the University of Minnesota that focused on leadership during resuscitations.^8^ Rather than focusing exclusively on resuscitation leadership, we designed a curriculum that additionally introduced individual leadership skills and leadership reflections, while also touching on team leadership concepts.

To ensure we were meeting the needs of our department’s residents, we performed a targeted needs assessment via discussion sessions with core educational faculty and our department’s education committee. Ultimately, we chose a longitudinal, progressive, seminar-based curriculum to introduce leadership concepts at key transition points in residents’ progression through the program.

### Goals of the curriculum

The goals of our leadership curriculum were to expose EM residents to the basics of leadership, to provide a graduated series of interactive, psychologically safe environments to explore individual leadership styles, to review interesting relevant literature, and to discuss leadership principles and experiences with senior leaders in our Emergency Department.

### Objectives of the curriculum

By the end of this curriculum, learners will be able to:

1. Understand the importance of deliberate leadership practice to success as an emergency physician.
2. Explain common leadership styles, their utility in certain clinical situations, and their relative benefits and drawbacks.
3. Understand specific behaviors and actions that can enhance or detract from a clinician’s leadership in the emergency department.

### Educational strategies

See curriculum chart.

### Results and tips for successful implementation

The curriculum was first implemented in 2017, and we have repeated it yearly since. We have found each session requires about one hour of preparation time and one hour to execute. We utilize a working group of five interested faculty and typically one to two interested senior residents each year to plan and execute the sessions. Approximately 60 emergency medicine residents per year participate in the activities as part of their normal residency conference curriculum.

Regarding resident satisfaction, after the pilot session (implemented in 2017), 89% of the approximately 50 residents who participated rated the workshops as either “useful” or “very useful” (4 or 5) on a 5-point Likert scale. The response rate for this survey was 100%, and the participation rate for the session was 81% of all residents.

After the 2020 curriculum concluded, we conducted another survey of the residents to re-assess their satisfaction with this curriculum. The survey included several questions on a 5-point Likert scale as well as the opportunity to provide suggestions for future content. Twenty-one of 60 residents responded (14 PGY-1, 4 PGY-2, 3 PGY-3). Ninety-one percent agreed or strongly agreed that the information presented was useful (remainder were neutral, none disagreed), and the same percentage agreed that they enjoyed the workshop. All respondents agreed or strongly agreed that leadership is an important topic for resident physicians. Seventy-six percent reported that the inclusion of leadership material improved their overall satisfaction with the residency curriculum.

Finally, we re-surveyed our PGY-1 and 2 classes after the 2021 curriculum concluded. The PGY3 class did not receive a workshop that year due to scheduling conflicts related to the COVID-19 pandemic. Fifteen of 30 attendees responded. As before, all strongly agreed (53%) or agreed (47%) that the workshop was useful. All strongly agreed (73% ) or agreed (27%) that it is important for resident physicians to learn about leadership. Fifty-three percent strongly agreed and 40% agreed that they enjoyed the leadership workshop, and the same proportion reported that this workshop improved their satisfaction with the overall residency curriculum. When asked for ideas for future workshop topics, the most requested topics included on-shift time management and how to become a better educator on-shift.

### Associated content

Appendix A – Curriculum Overview and Discussion Points

Appendix B – PGY1 Session Slideshow

Appendix C – PGY2 Session Slideshow

### Evaluation and feedback

Early feedback included a desire to discuss transitions in leadership during yearly residency promotions, as well as how to handle conflict with hospital staff when disagreements or negative interactions arise. In response to this, we included additional discussion in the PGY-1 curriculum on handling conflict with nurses and consultants during the discussion part of the seminar.

Initially, we held the curriculum in the middle of the academic year. Based on the feedback to focus on leadership transitions during promotion, we changed the implementation timeframe to the April–May period to better align with the resident promotion schedule. Subsequent feedback on this change was positive, and the curriculum remains scheduled for springtime each year.

After utilizing this curriculum for seven years, we feel that it is best implemented when tailored to the residents’ experiences and struggles within their learning environment. While many leadership practices are universal, our residents’ challenges at an urban safety-net hospital may be different from those in other settings. Educators should consider the unique culture and challenges of their institution when designing the discussion points.

## Figures and Tables

**Table t2-jetem-9-1-c1:** 

Topic	Recommended Educational Strategy	Educational Content	Objectives	Learners	Timing, Resources Needed	Recommended Assessment, Milestones Addressed
Introduction to Leadership	“Lifting Sticks” introductory activity to discuss natural leadership and group dynamics Short presentation on resuscitation leadership Small group discussion on on-shift leadership principles and best practices	- ACLS best practices for resuscitation leadership - Data on resuscitation leadership and outcomes - Overview of common leadership styles	The learner will understand the importance of on-shift leadership, the common types of leadership styles, and best practices for and challenges to on-shift leadership	PGY-1 (best done when approaching promotion to PGY2)	Space required: Small lecture hall or small group room (sufficient for PGY-1 class size) 10 minutes: “Lifting Sticks Activity” - Requires lightweight gardening stakes approx. 2–3 feet long, can be purchased at local home improvement store - Instructions in Appendix A 15 minutes: Discussion of Resuscitation Leadership - Suggested presentation slides in Appendix B - Ford K, Menchine M, Burner E, et al. Leadership and teamwork in trauma and resuscitation. *Western Journal of Emergency Medicine*. 2016; 17(5):549–556. 10 minutes: Overview of Common Leadership Styles - Suggested presentation slides in appendix B - Eagly AH, Johannesen-Schmidt MC, van Engen ML. Transformational, transactional, and laissez-faire leadership styles: a meta-analysis comparing women and men. *Psychol Bull*. 2003;129(4):569–591. 20 minutes: Group Discussion of leadership challenges and best practices on shift - Discussion points in Appendix A	Milestones: PBLI2, PROF1, ICS2 Assessment: Post-class survey, direct observation on shift
Senior Resident Advanced Leadership Skills	Small group presentation on advanced leadership skills and styles Discussion of high-efficiency practices in the ED Open discussion on learners’ experiences with best practices for on-shift leadership	- Review of leadership styles and general principles - Introduction to high-efficiency clinical practices and correlation to leadership - Pearls and pitfalls for on-shift leadership	The learner will understand the relationship between leadership and efficiency on-shift. The learner will reflect on their own leadership style and performance thus far in residency.	PGY-2 (best done when approaching promotion to PGY3)	Space required: small group room or classroom sufficient for your residency class size 15 minutes: Advanced Leadership Styles discussion - Curriculum Overview in Appendix A - Discussion slides in Appendix C 15 minutes: High-Efficiency Practices in the ED - Discussion slides in Appendix C - See also: Bobb MR, Ahmed A, Van Heukelom P, et al. Key high-efficiency practices of emergency department providers: a mixedmethods study. *Acad Emerg Med*. 2018 Jul;25(7):795–803.. Epub 2018 Jan 22. PMID: 29265539. doi: 10.1111/acem.13361 20 minutes: Open discussion and reflection on leadership experience from past year	Milestones: PBLI2, PROF1, ICS2 Assessment: Post-class survey, participation in reflective discussion, direct observation on shift
Preparing for Leadership Post-Residency	Reflection on leadership journey in residency Panel discussion on leadership during transition to attending physician and future formal leadership opportunities	- Experienced attendings and local leaders providing their leadership perspectives	The learner will reflect on their leadership journey and anticipate future leadership opportunities and challenges	Residents nearing graduation (PGY3 or 4)	Space required: small group room or classroom sufficient for your residency class size 15 minutes: Personal reflections and discussions - Specific Instructions in Appendix A 40 minutes: Panel discussion on Transition to Attending Leadership Role and Future Leadership Opportunities - 3–4 panel members: departmental leaders (chair, vice chair, medical director, residency director, leader within national org, etc). - Discussion points in Appendix A	Milestones: PBLI2 Assessment: Post-class survey or reflective piece, participation in reflective discussion

## DIDACTICS AND HANDS-ON CURRICULUM

### Curriculum Chart

[Table t2-jetem-9-1-c1]

## Appendix A Curriculum Overview

### PGY-1 Session (1 hour)

- 10–15 minutes: Ice breaker – “Lifting Sticks”
  ○ Using lightweight gardening stakes approximately 2–3 feet long, groups of learners must each support the stake using 1 finger. Each learner’s finger is evenly spaced along the stick. The group must then raise the stick above their heads and lower it back to the ground, while everyone’s finger maintains contact with the stick at all times.
  ○ Discussion afterward: How did the group coordinate to accomplish this? Did a leader emerge? Which worked better: having a leader direct the group, or everyone communicating simultaneously?
- 15–20 minutes: Discussion of Resuscitation Leadership
  ○ Learners are encouraged to share experiences with good or difficult examples of resuscitation leadership and to identify best practices.
  ○ Review ACLS best practices for resuscitation leadership
    ▪ Mutual respect for team members
    ▪ Clear roles and responsibilities
    ▪ Clear messages or instructions
    ▪ Read back instructions
    ▪ Constructive intervention to prevent errors
    ▪ Eliciting ideas (“knowledge sharing”)
    ▪ Openness to constructive criticism and performing after-event debriefing
  ○ Review data on resuscitation leadership and outcomes
    ▪ Ford K, Menchine M, Burner E, et al. Leadership and Teamwork in Trauma and Resuscitation. *Western Journal of Emergency Medicine*. 2016; 17(5):549–556.
    ▪ Effective leadership associated with better processes of care in both trauma and medical resuscitations.
    ▪ Teams with strong leaders more likely to adhere to ATLS, decreased time to CT, intubation, and transfer to OR, more successful cardiopulmonary resuscitations.
    ▪ Poor leadership was associated with worse outcomes and performance on these same measures.
- Remainder of time (20–25 minutes): Discuss Transition to Leadership Role in ED
  ○ Overview of common leadership styles
    ▪ Eagly AH, Johannesen-Schmidt MC, van Engen ML. Transformational, transactional, and laissez-faire leadership styles: a meta-analysis comparing women and men. *Psychol Bull*. 2003;129(4):569–591.
    ▪Transformational Leadership
      - Qualities that motivate respect and pride among those associated with the leaders
      - Communicates values, purpose, and importance of the mission
      - Exhibits optimism and excitement
      - Examines new perspectives for solving problems and completing tasks
      - Focuses of development and mentorship
    ▪ Transactional
      - Provides rewards for satisfactory performance by followers
      - Rather than proactively preventing problems or maintaining standards, attends to mistakes or failures after they occur, may wait until problems become severe before attending to them
    ▪ Laissez-Faire
      - Absence and lack of involvement during critical junctures
    ▪ Discussion questions
      - Which type of leader do you prefer to work for? Which is most effective?
      - What type of leader are you, on shift?
      - How do you become the leader you want to be?
  ○ Group discussion of leadership challenges and best practices on-shift
    ▪ Managing, communicating, and establishing relationships with staff
    ▪ Working with other departmental leaders, such as the charge nurse
    ▪ Handling disagreements with others
    ▪ Managing difficult communications (including dissatisfied patients or family, difficult conversations with consultants)
    ▪ Discussing stressors that detract from our leadership (volume, acuity, personal stress, fatigue, etc) and how to mitigate these
  ○ (If Time) Encourage residents to take a moment to think about their leadership role models in the department
    ▪ May be a fellow resident, attending, RN
    ▪ What is it about their leadership style that they admire?
    ▪ What steps can the learner take to adopt that leadership style?

### PGY-2 Session (1 hour)

- 10 minutes: Leadership Styles Discussion
  ○ Everyone has a default style, but there exists a need to occasionally modify our style for the situation
  ○ Have the learners reflect:
    ▪ What type of leader are you on shift?
    ▪ Have you incorporated any changes to your leadership since the last session as a PGY1?
    ▪ Does your leadership style change in different situations? Should it? When does it?
  ○ Directive Leadership in resuscitations
  ○ Empowering Leadership in lower acuity situations
  ○ Collaborative Leadership with consultants
- 15 minutes: Discussion of High-Efficiency Practices in the ED
  ○ Elicit learners’ perspectives on how to improve efficiency
    ▪ Link their efficiency practices to leadership best practices
  ○ Review findings from the paper cited below:
    ▪ Emphasis on the leadership elements (use people’s names, “run the board,” have conversations with staff)
    ▪ Bobb MR, Ahmed A, Van Heukelom P, et al. Key High-efficiency Practices of Emergency Department Providers: A Mixed-methods Study. *Acad Emerg Med.* 2018 Jul;25(7):795–803. Epub 2018 Jan 22. PMID: 29265539. doi: 10.1111/acem.13361
- 10 minutes: Developing leadership values
  ○ First exercise: Run through four questions:
    ▪ What do I believe about leadership?
    ▪ What can you expect of me?
      - As a leader, what is your commitment to those whom you lead?
    ▪ What can I expect of you?
      - What behaviors or standards should you expect from those you lead?
    ▪ What will attract negative attention?
      - What behaviors might I exhibit that could be harmful to my leadership goals and values?
  ○ Second Exercise: Identify values
    ▪ Have learners review the list of leadership values and select the three that are most important to them
      - Have them reflect on why they chose those values and identify what behaviors communicate these values to others
- Remainder of time (20–25 minutes): Open discussion
  ○ Challenges and Best Practices in ED Leadership
    ▪ Allow residents to reflect on challenges to leadership
      - Personal
      - Situational
      - Cultural within the department
      - Specific struggles within the department
    ▪ How do we respond when the department is “on fire”? How do we manage our and others’ emotions?
    ▪ How do we balance our need to advocate for patient care and occasionally push for a task to be completed while remaining team-oriented and collegial?
    ▪ How do we lead when we don’t agree with a particular institutional or departmental initiative? How does our personal feeling about the initiative impact our leadership?
  ○ Reflect on discussion from PGY1 year – what elements did you incorporate? What worked and what did not work?

### PGY-3 Session (1 hour)

#### Goals

- 15 minutes: Personal Reflections and Discussions
  ○ Learners reflect on a personal leadership challenge they encountered in the ED
    ▪ What was the specific challenge? (ie, listening to others, communicating thoughts, validating others’ ideas, etc)
    ▪ What was the outcome?
    ▪ Were there external factors contributing to the challenge? (busy shift, fatigue, etc)
  ○ Discuss with a partner and identify ways to improve next time
- Remainder of time (40–45 minutes): Panel Discussion on Transition to Attending Leadership Role and Additional Departmental Leadership Roles
  ○ 3–4 panel members - leaders from the department
    ▪ Seasoned attendings or faculty
    ▪ Medical director
    ▪ Department chair
    ▪ Residency/Clerkship director
    ▪ Hospital leadership
  ○ Discussion points:
    ▪ What specific steps do you take to establish your role as a leader in the ED when you start a shift?
    ▪ Who are your role models when you think about being a leader in the ED?
    ▪ (If they have a formal leadership role outside of clinical shifts): Do you use the same leadership styles on-shift and in your other leadership role? If not, how do they differ?
    ▪ (For earlier-career attendings on the panel): Reflect on your transition to becoming an attending or starting in a new department. What challenges did you face from a leadership standpoint?

## Appendix B PGY 1 Session Slideshow

Please see associated Power Point

## Appendix C PGY 2 Session Slideshow

Please see associated Power Point
