# Supplementary material for: A Novel Leadership Curriculum for Emergency Medicine Residents
Source: J Educ Teach Emerg Med. 2024 Jan 31;9(1):C1–C15. doi: 10.21980/J81D2S (PMC10854878; doi:10.21980/J81D2S)
Supplement: Supplementary file 1 — Please see associated Power Point [file jetem-9-1-C1-AppendixB.pptx]

## Slide 1
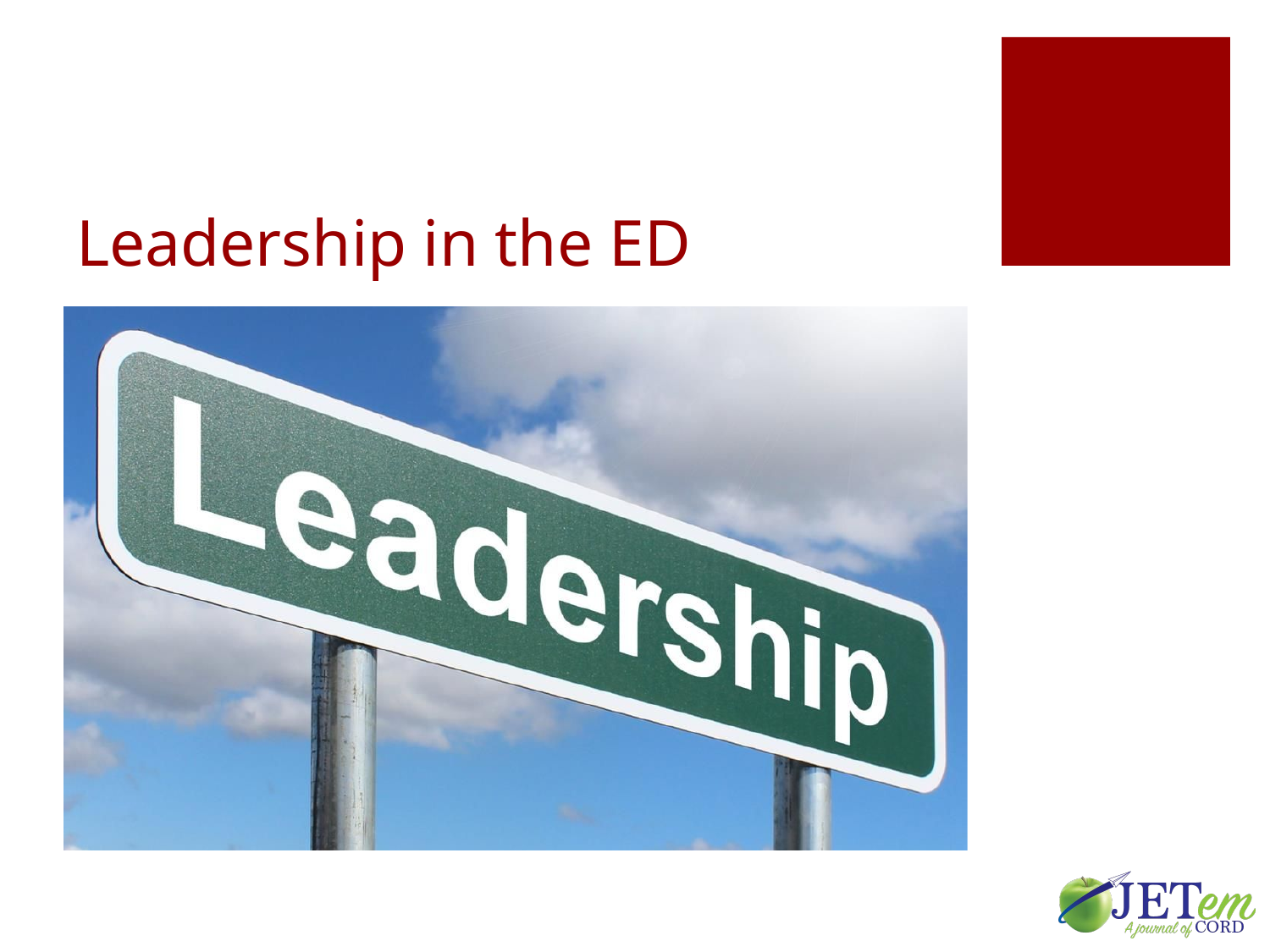

# Leadership in the ED

## Slide 2
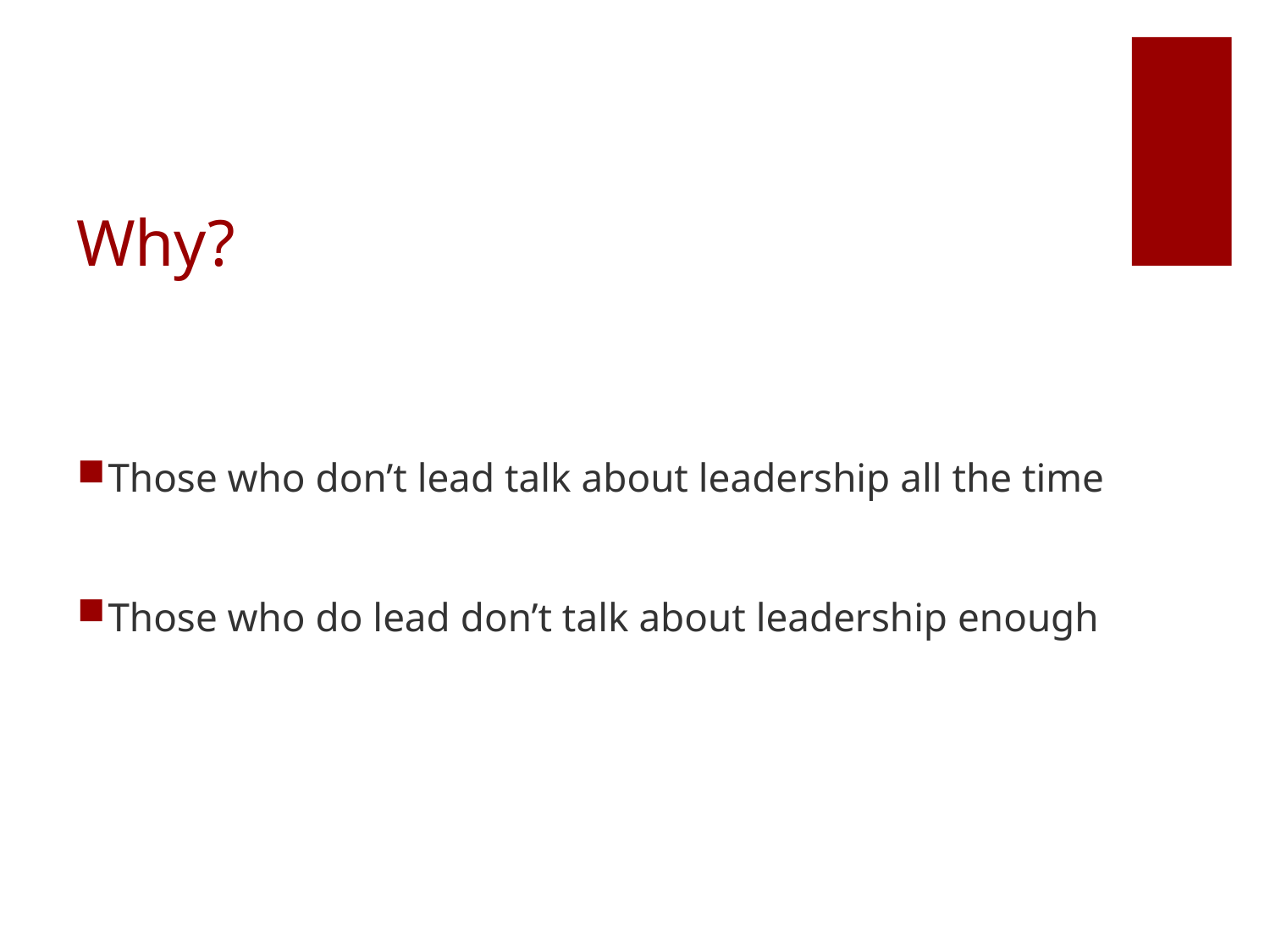

# Why?
Those who don’t lead talk about leadership all the time
Those who do lead don’t talk about leadership enough

## Slide 3
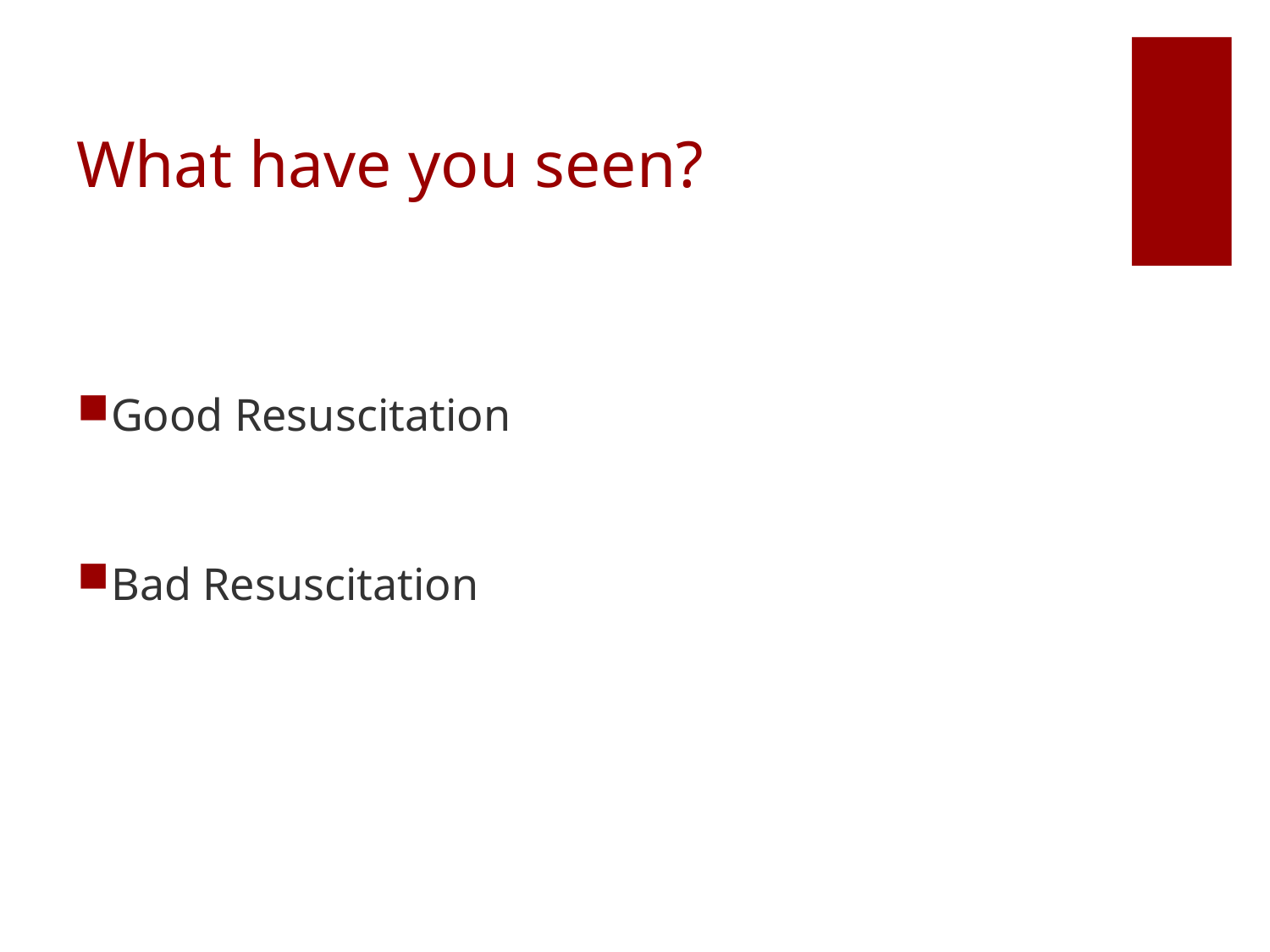

# What have you seen?
Good Resuscitation
Bad Resuscitation

## Slide 4
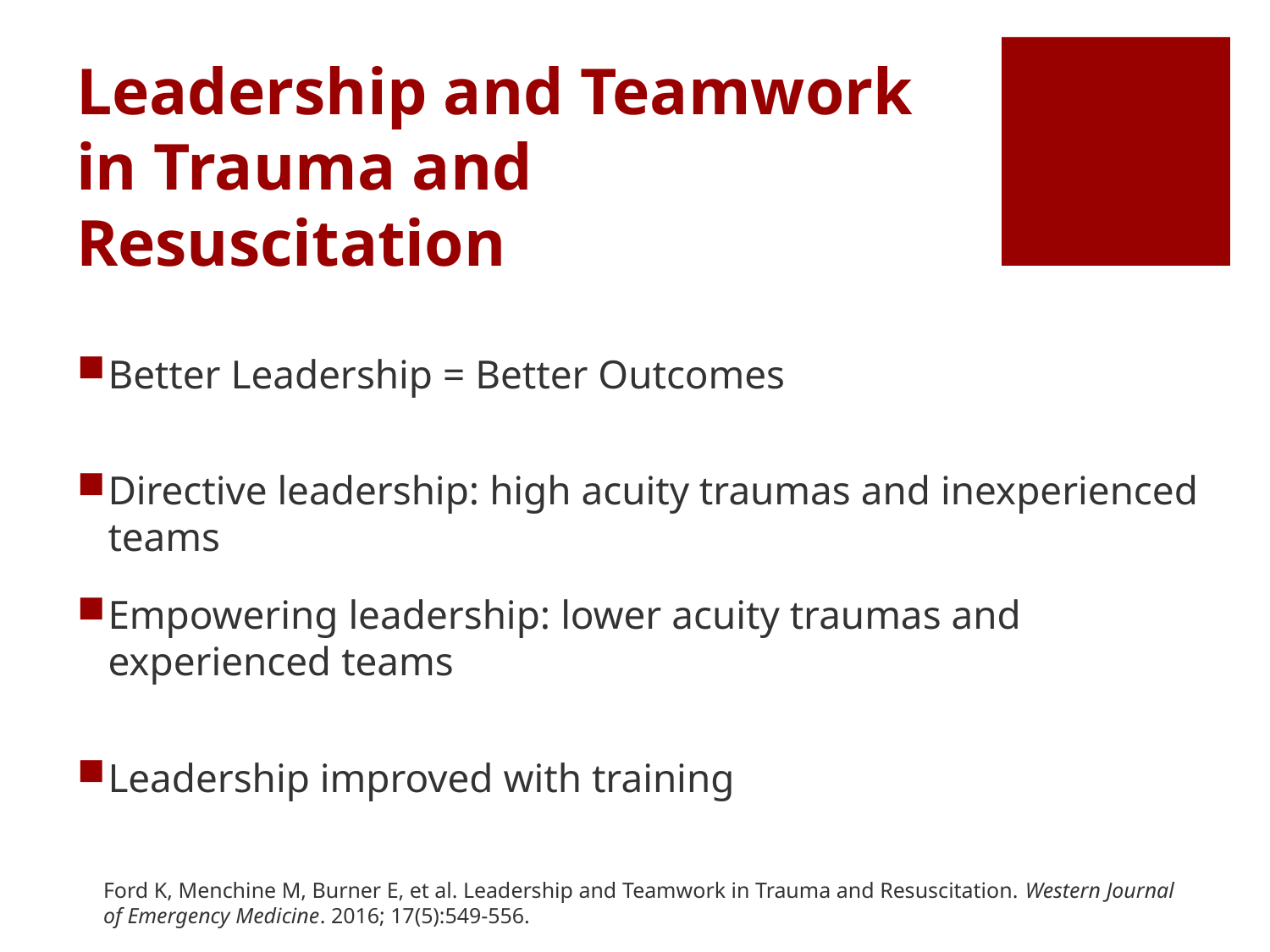

# Leadership and Teamwork in Trauma and Resuscitation
Better Leadership = Better Outcomes
Directive leadership: high acuity traumas and inexperienced teams
Empowering leadership: lower acuity traumas and experienced teams
Leadership improved with training
Ford K, Menchine M, Burner E, et al. Leadership and Teamwork in Trauma and Resuscitation. Western Journal of Emergency Medicine. 2016; 17(5):549-556.

## Slide 5
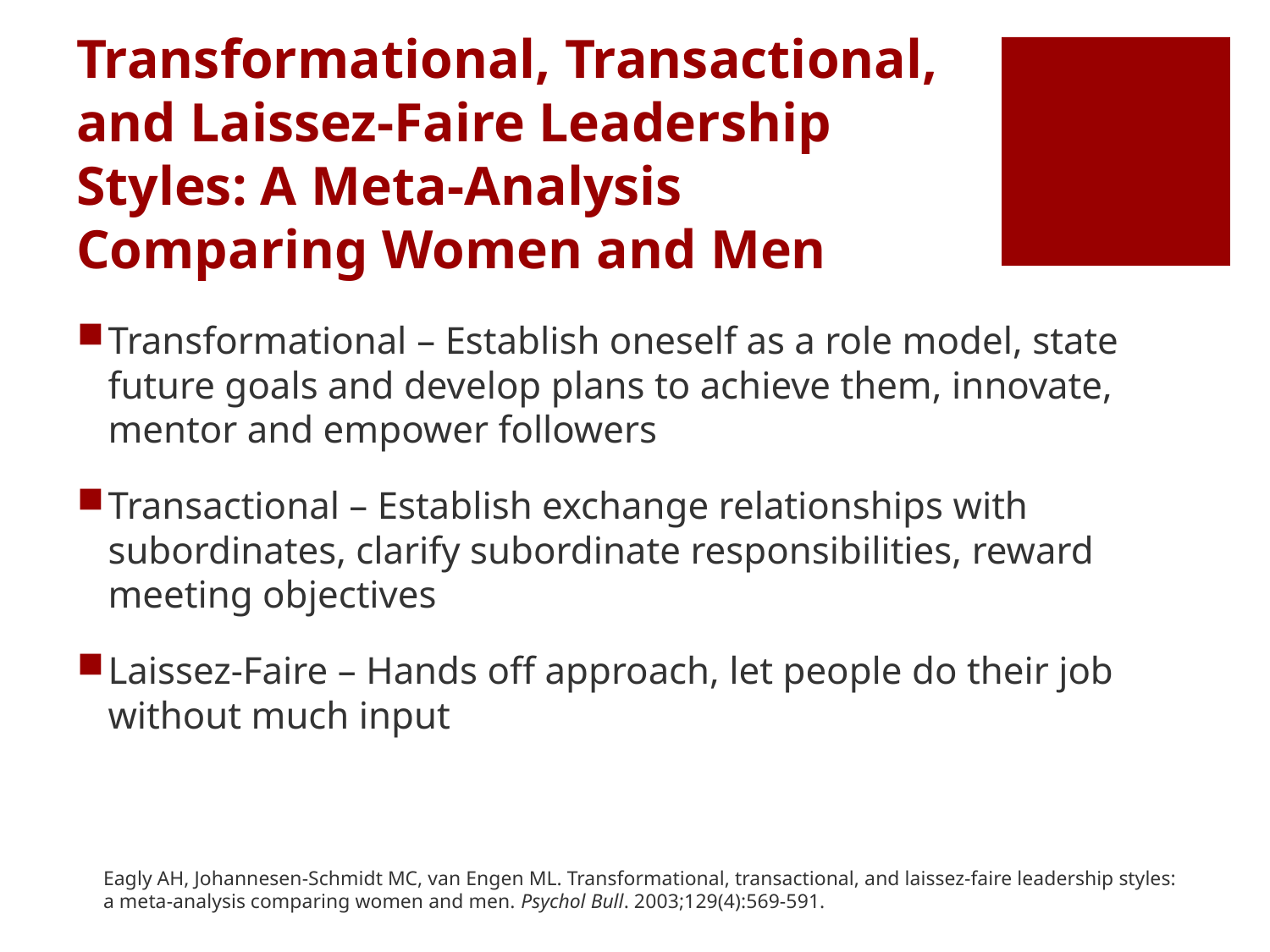

# Transformational, Transactional, and Laissez-Faire Leadership Styles: A Meta-Analysis Comparing Women and Men
Transformational – Establish oneself as a role model, state future goals and develop plans to achieve them, innovate, mentor and empower followers
Transactional – Establish exchange relationships with subordinates, clarify subordinate responsibilities, reward meeting objectives
Laissez-Faire – Hands off approach, let people do their job without much input
Eagly AH, Johannesen-Schmidt MC, van Engen ML. Transformational, transactional, and laissez-faire leadership styles: a meta-analysis comparing women and men. Psychol Bull. 2003;129(4):569-591.

## Slide 6
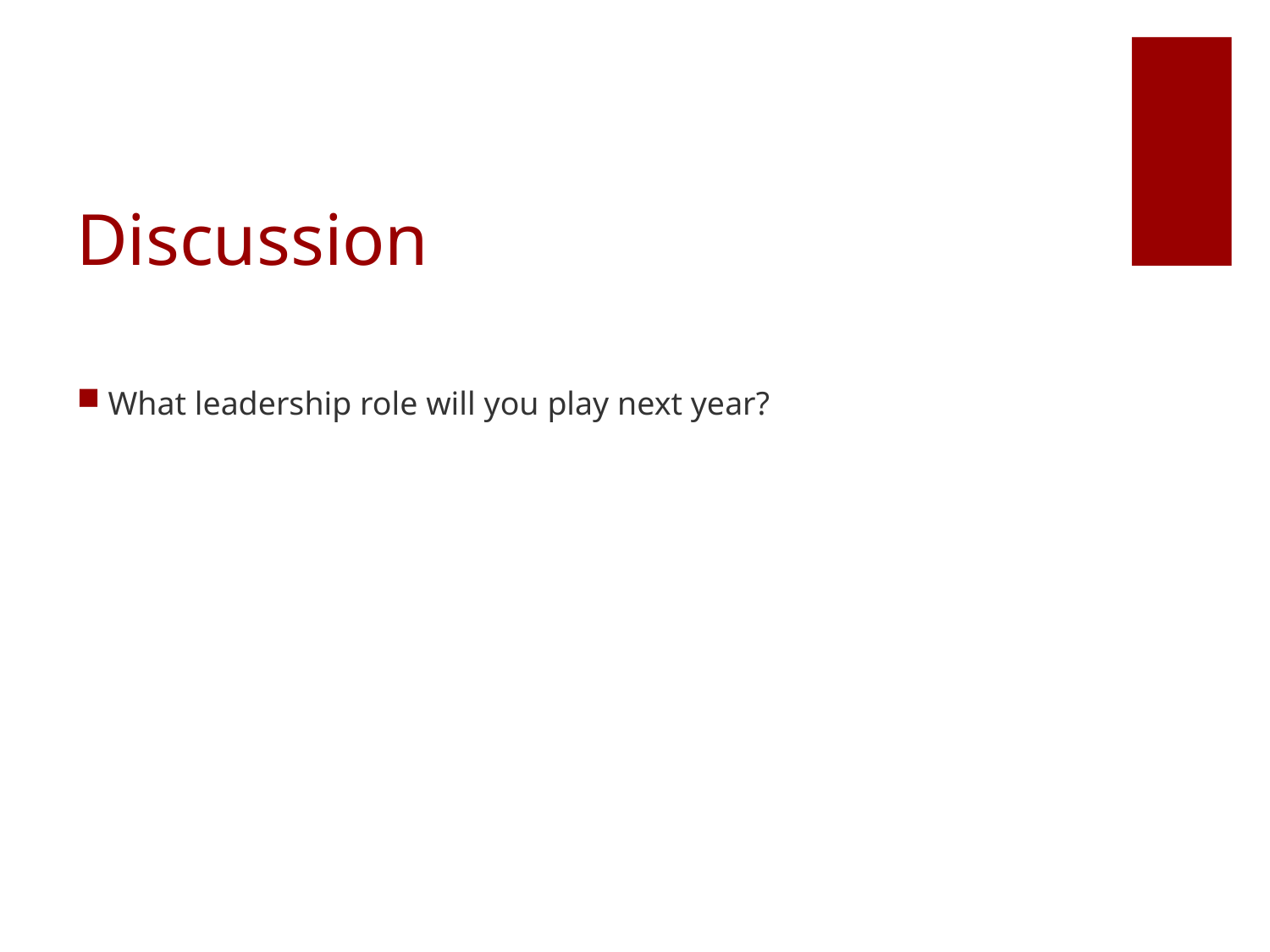

# Discussion
What leadership role will you play next year?

## Slide 7
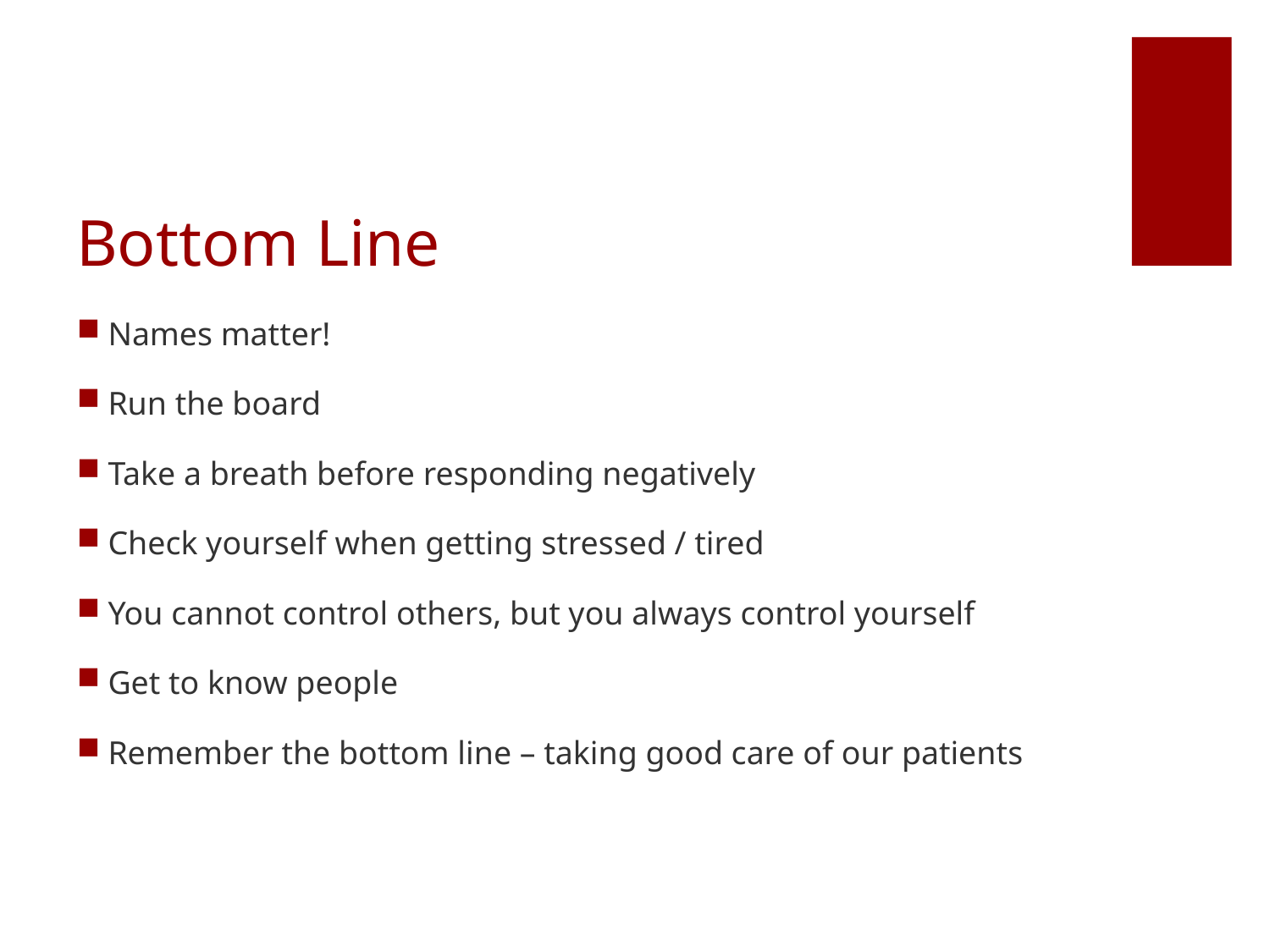

# Bottom Line
Names matter!
Run the board
Take a breath before responding negatively
Check yourself when getting stressed / tired
You cannot control others, but you always control yourself
Get to know people
Remember the bottom line – taking good care of our patients

## Slide 8
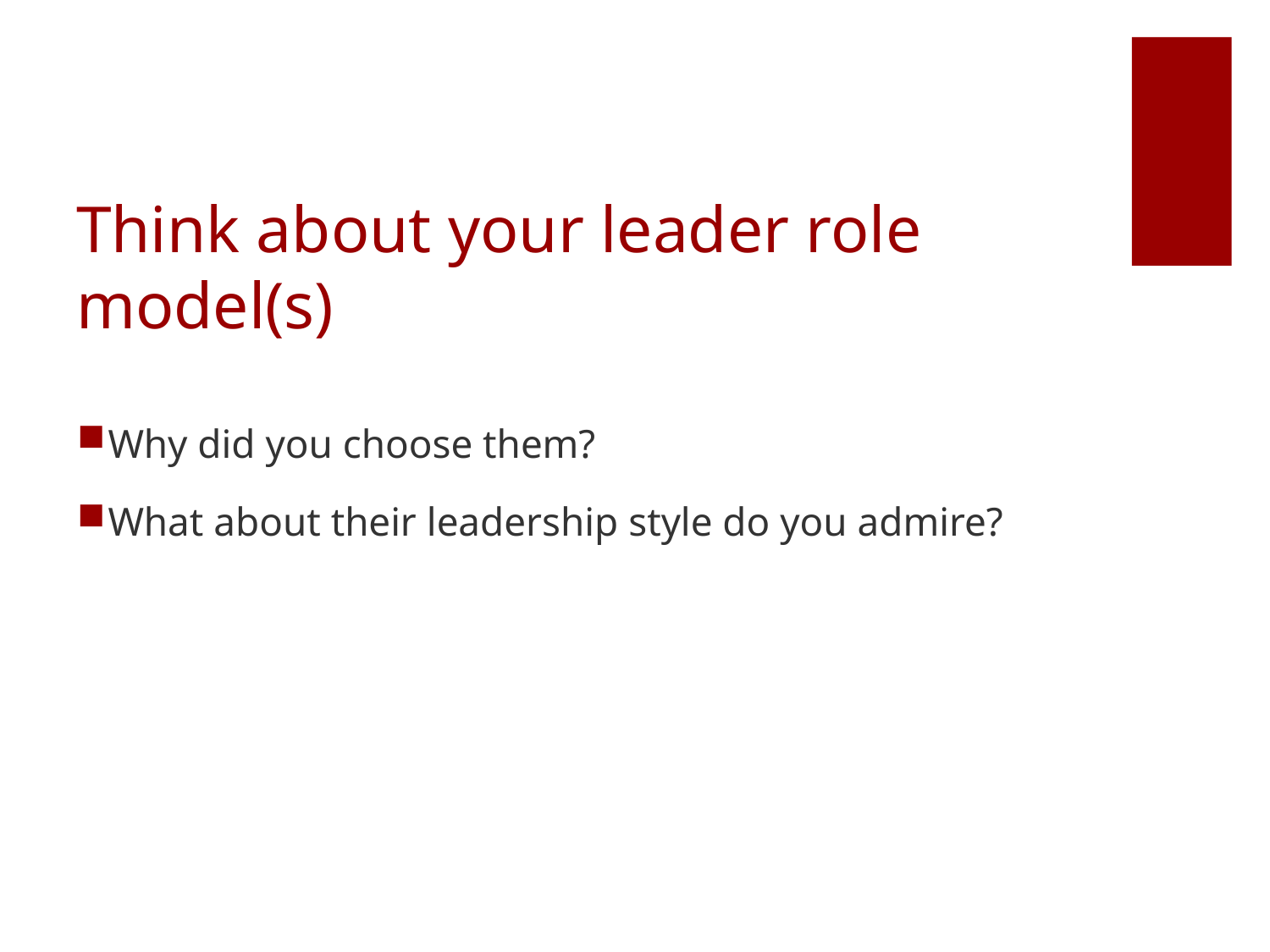

# Think about your leader role model(s)
Why did you choose them?
What about their leadership style do you admire?

## Slide 9
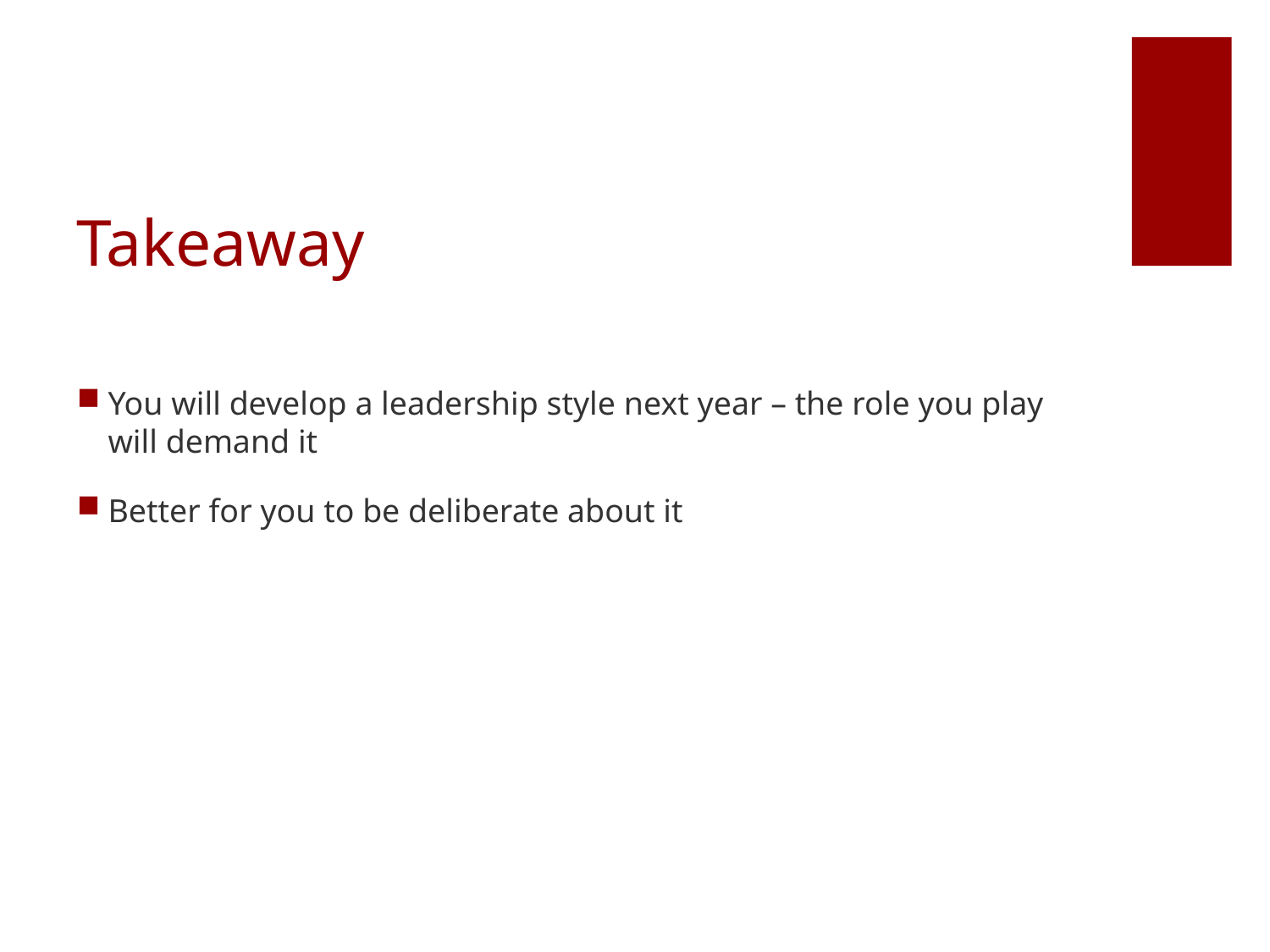

# Takeaway
You will develop a leadership style next year – the role you play will demand it
Better for you to be deliberate about it
