# Supplementary material for: A Novel Leadership Curriculum for Emergency Medicine Residents
Source: J Educ Teach Emerg Med. 2024 Jan 31;9(1):C1–C15. doi: 10.21980/J81D2S (PMC10854878; doi:10.21980/J81D2S)
Supplement: Supplementary file 2 — Please see associated Power Point [file jetem-9-1-C1-AppendixC.pptx]

## Slide 1
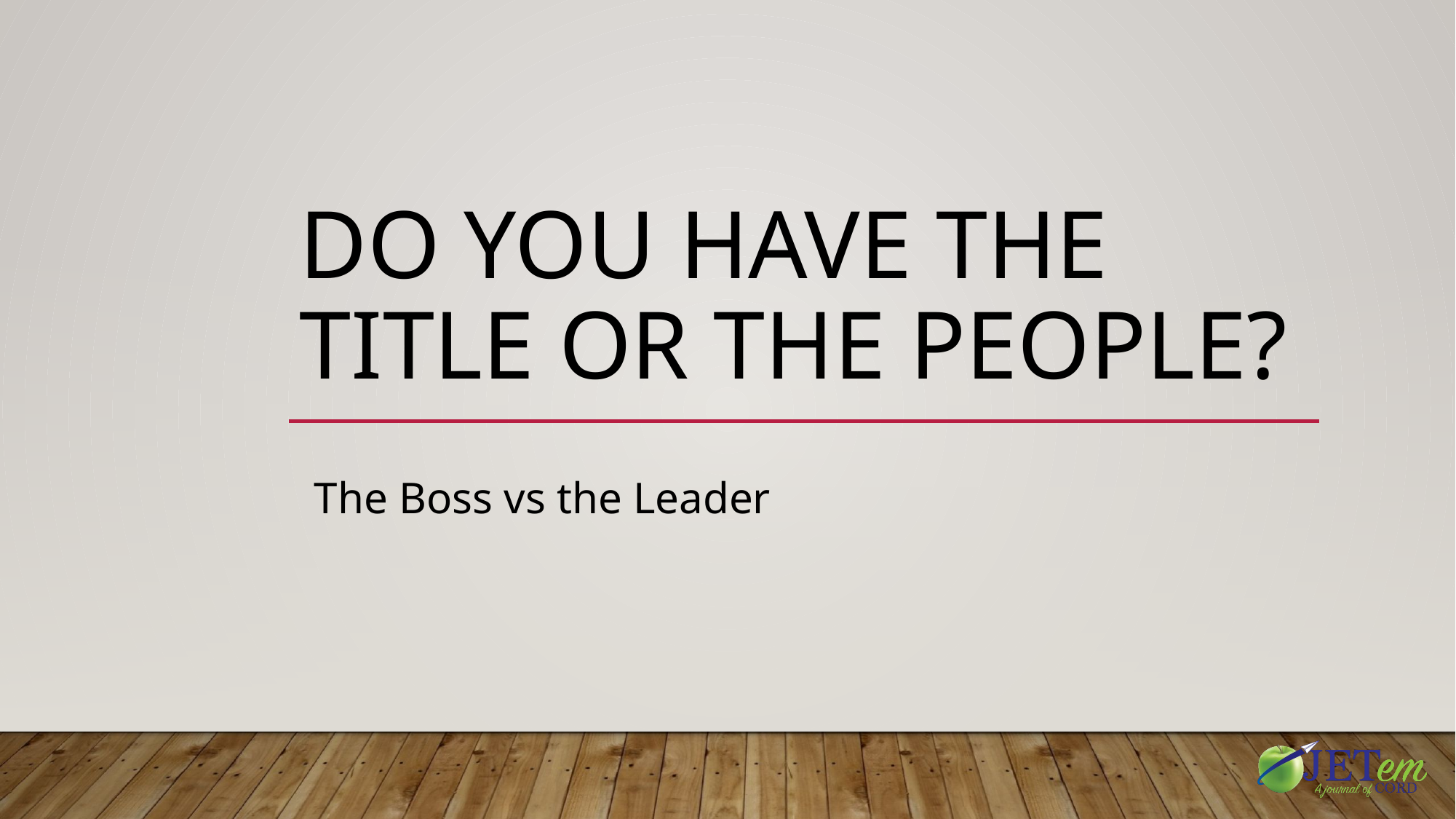

# Do you have the title or the people?
The Boss vs the Leader

## Slide 2
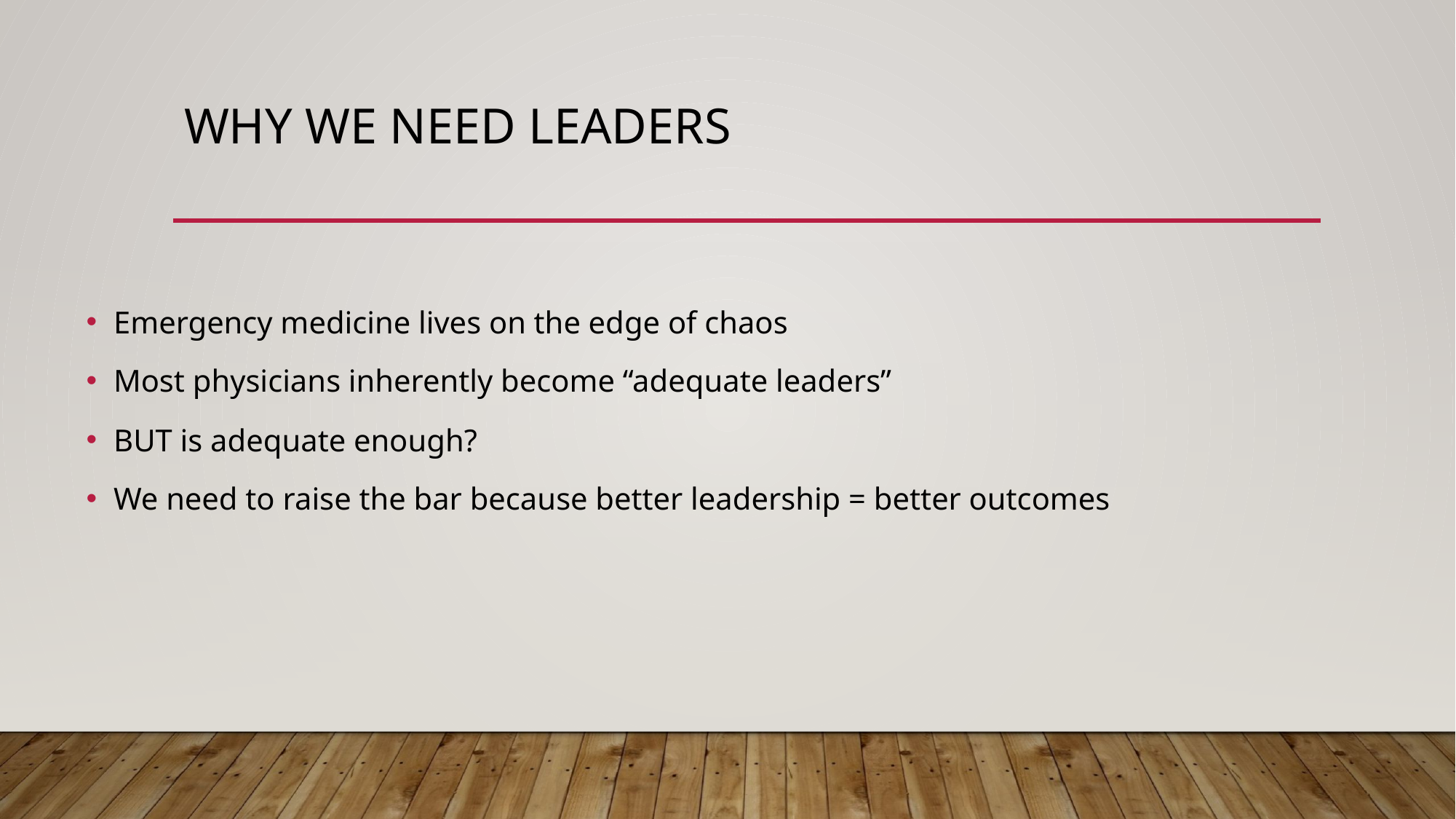

# Why we need leaders
Emergency medicine lives on the edge of chaos
Most physicians inherently become “adequate leaders”
BUT is adequate enough?
We need to raise the bar because better leadership = better outcomes

## Slide 3
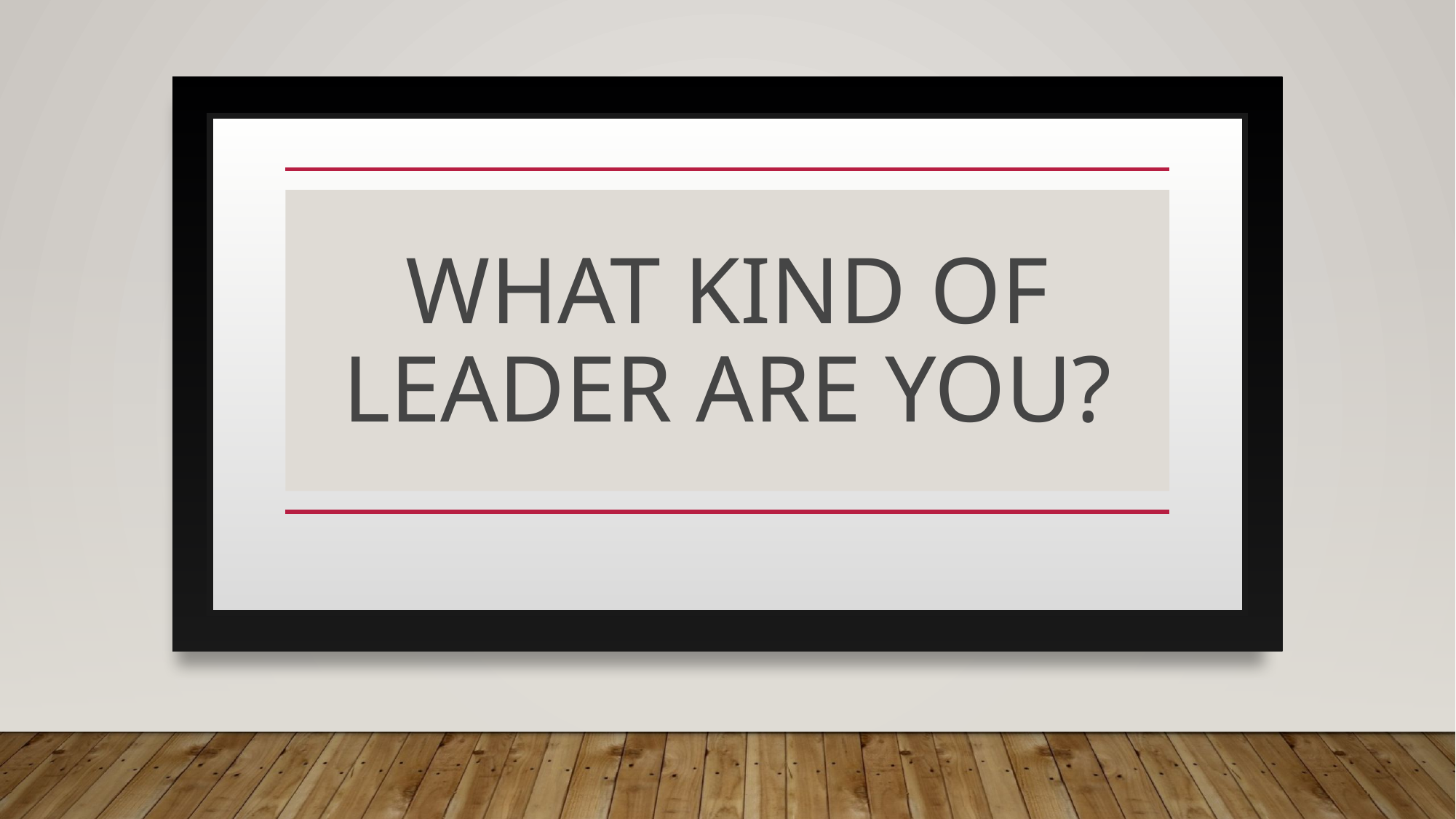

# What Kind of leader are you?

## Slide 4
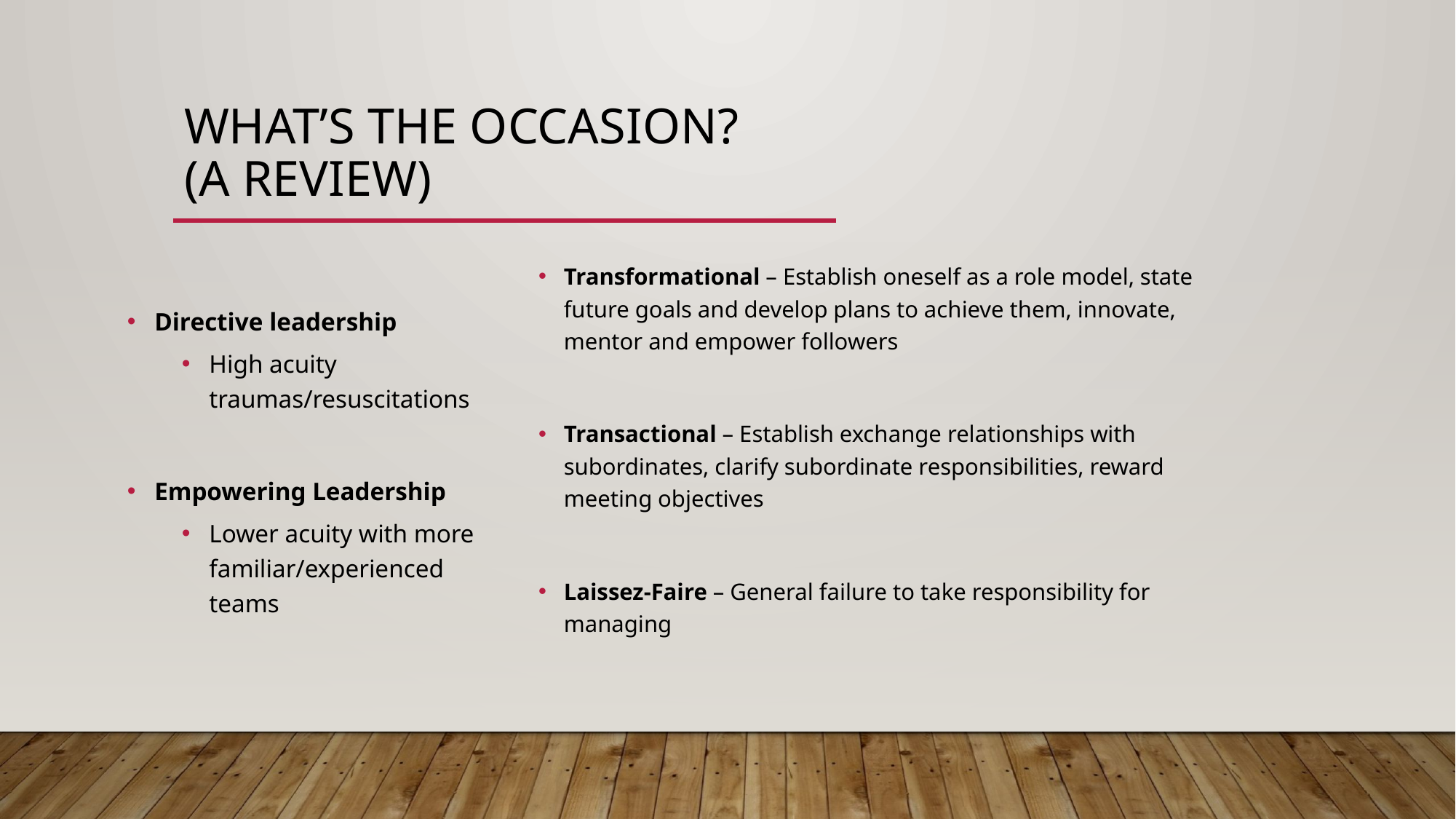

# What’s the occasion? (A review)
Transformational – Establish oneself as a role model, state future goals and develop plans to achieve them, innovate, mentor and empower followers
Transactional – Establish exchange relationships with subordinates, clarify subordinate responsibilities, reward meeting objectives
Laissez-Faire – General failure to take responsibility for managing
Directive leadership
High acuity traumas/resuscitations
Empowering Leadership
Lower acuity with more familiar/experienced teams

## Slide 5
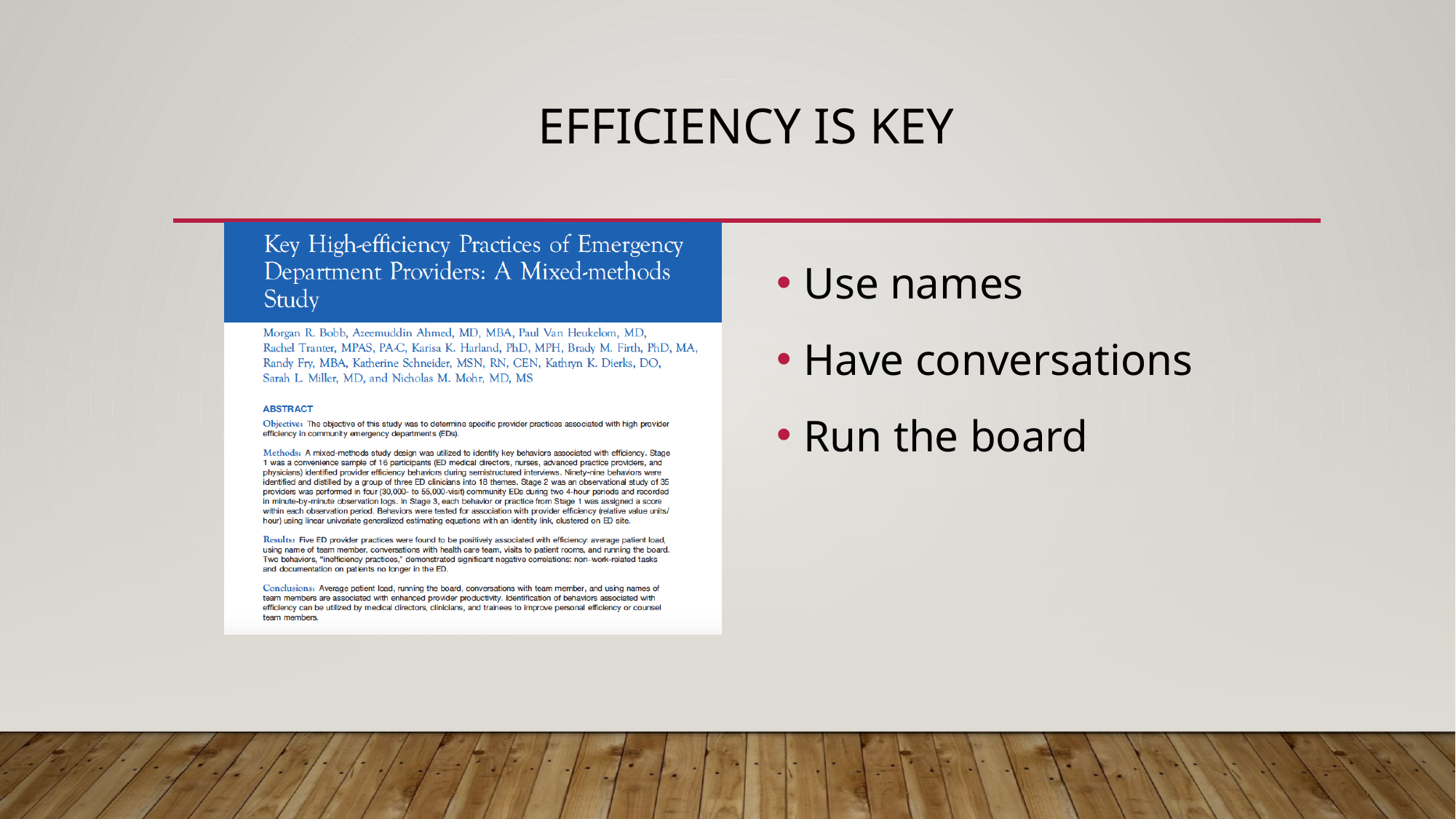

# Efficiency is Key
Use names
Have conversations
Run the board

## Slide 6
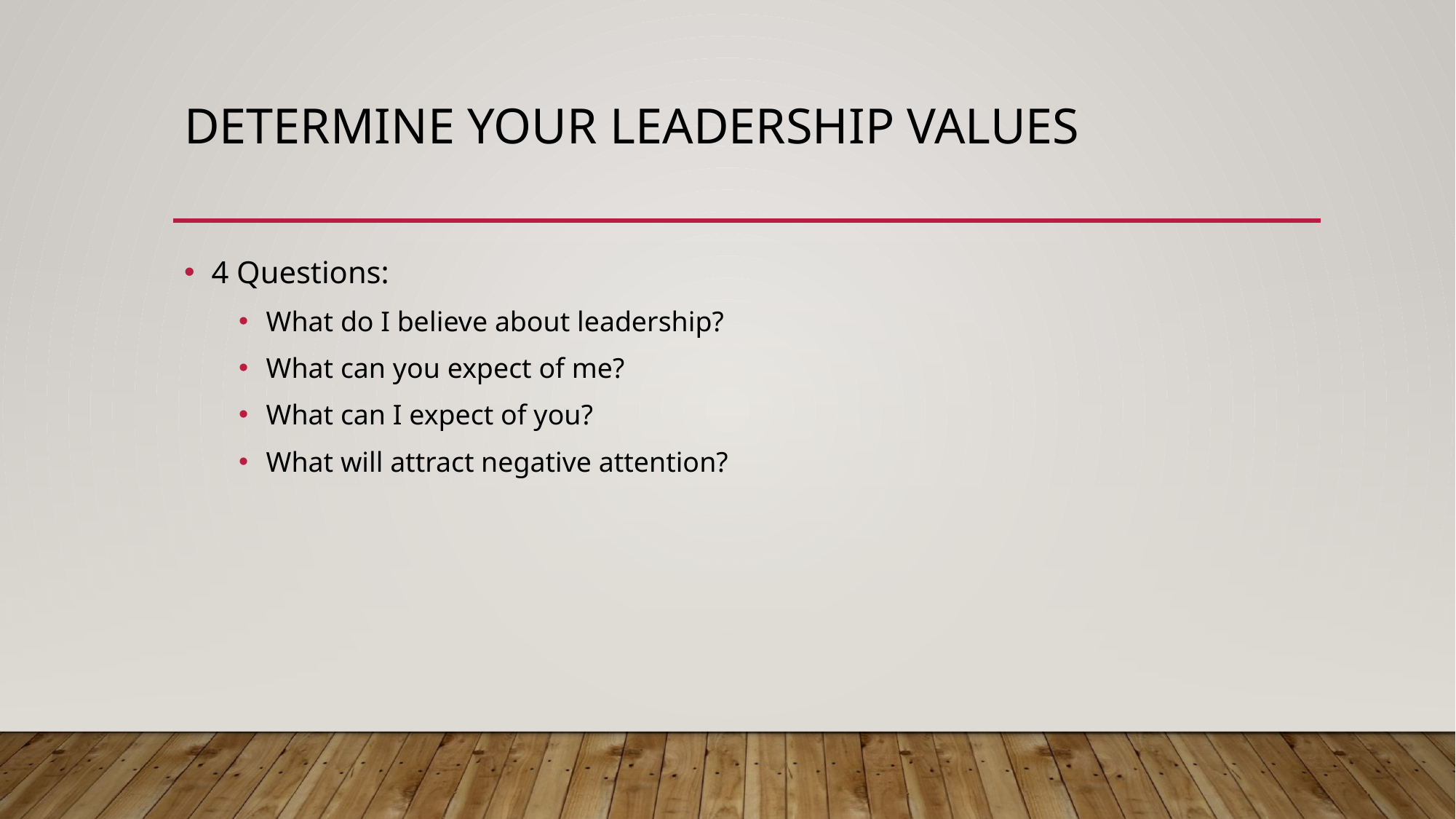

# Determine your leadership values
4 Questions:
What do I believe about leadership?
What can you expect of me?
What can I expect of you?
What will attract negative attention?

## Slide 7
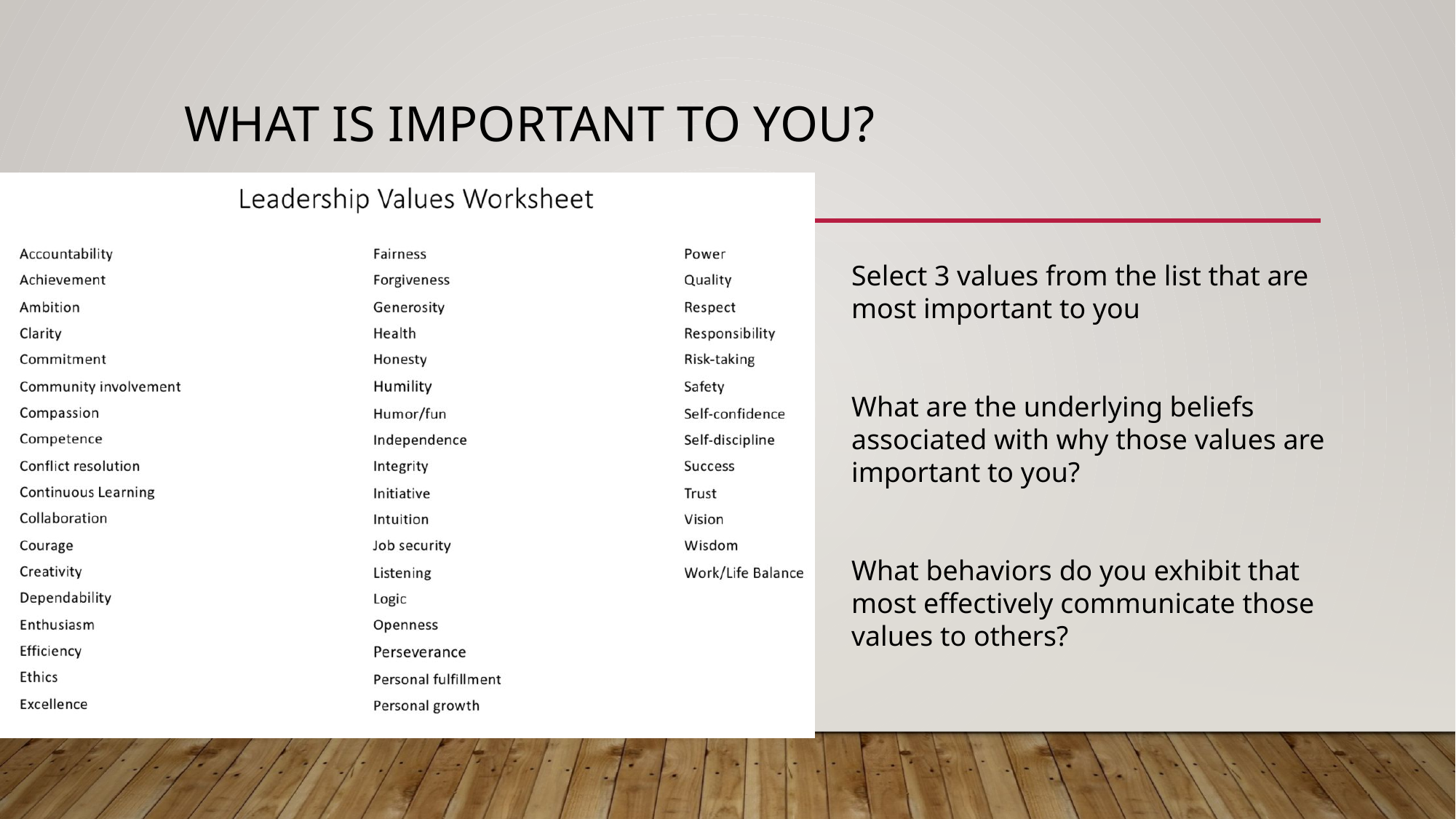

# What is important to you?
Select 3 values from the list that are most important to you
What are the underlying beliefs associated with why those values are important to you?
What behaviors do you exhibit that most effectively communicate those values to others?

## Slide 8
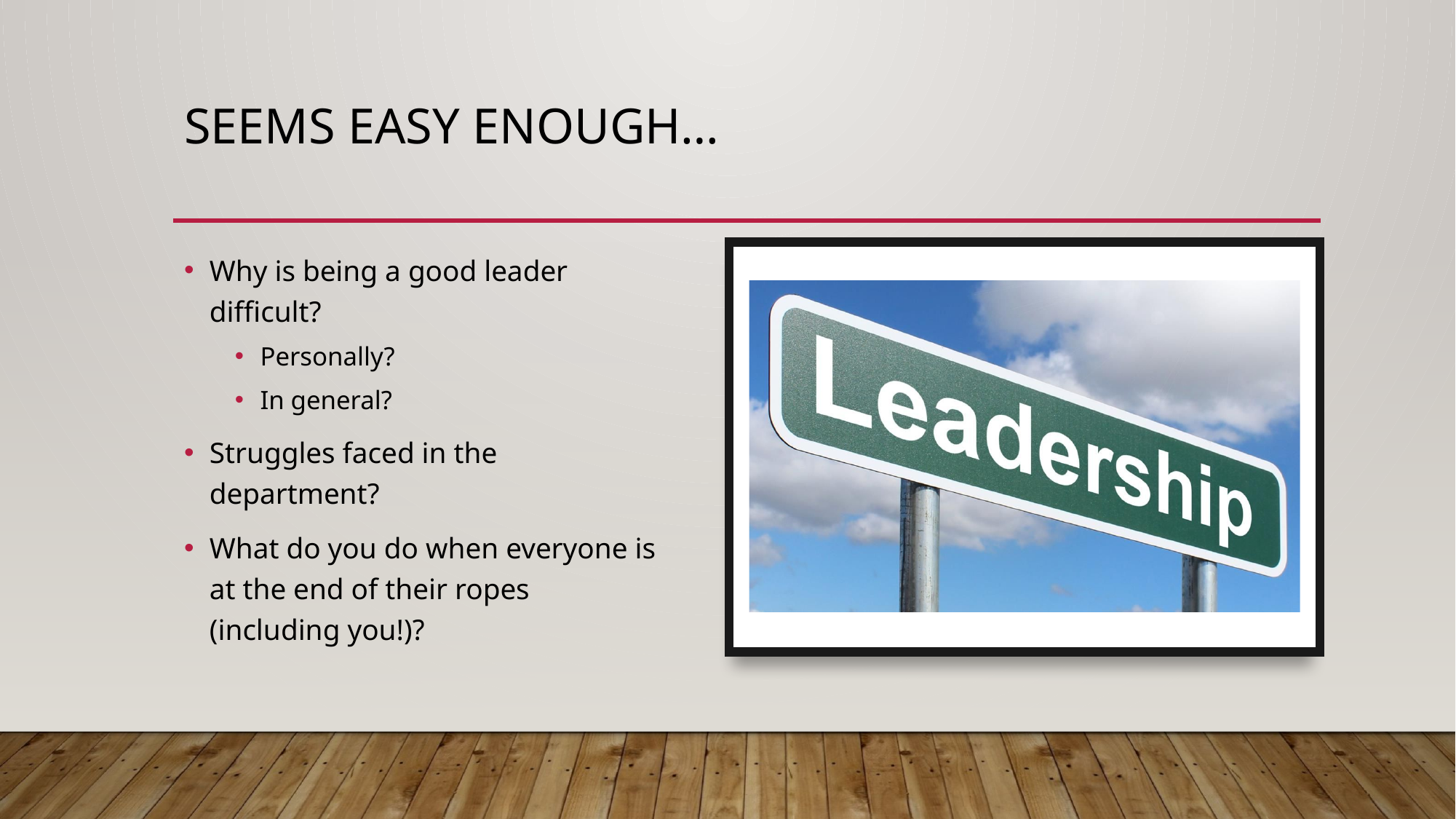

# Seems easy enough…
Why is being a good leader difficult?
Personally?
In general?
Struggles faced in the department?
What do you do when everyone is at the end of their ropes (including you!)?

## Slide 9
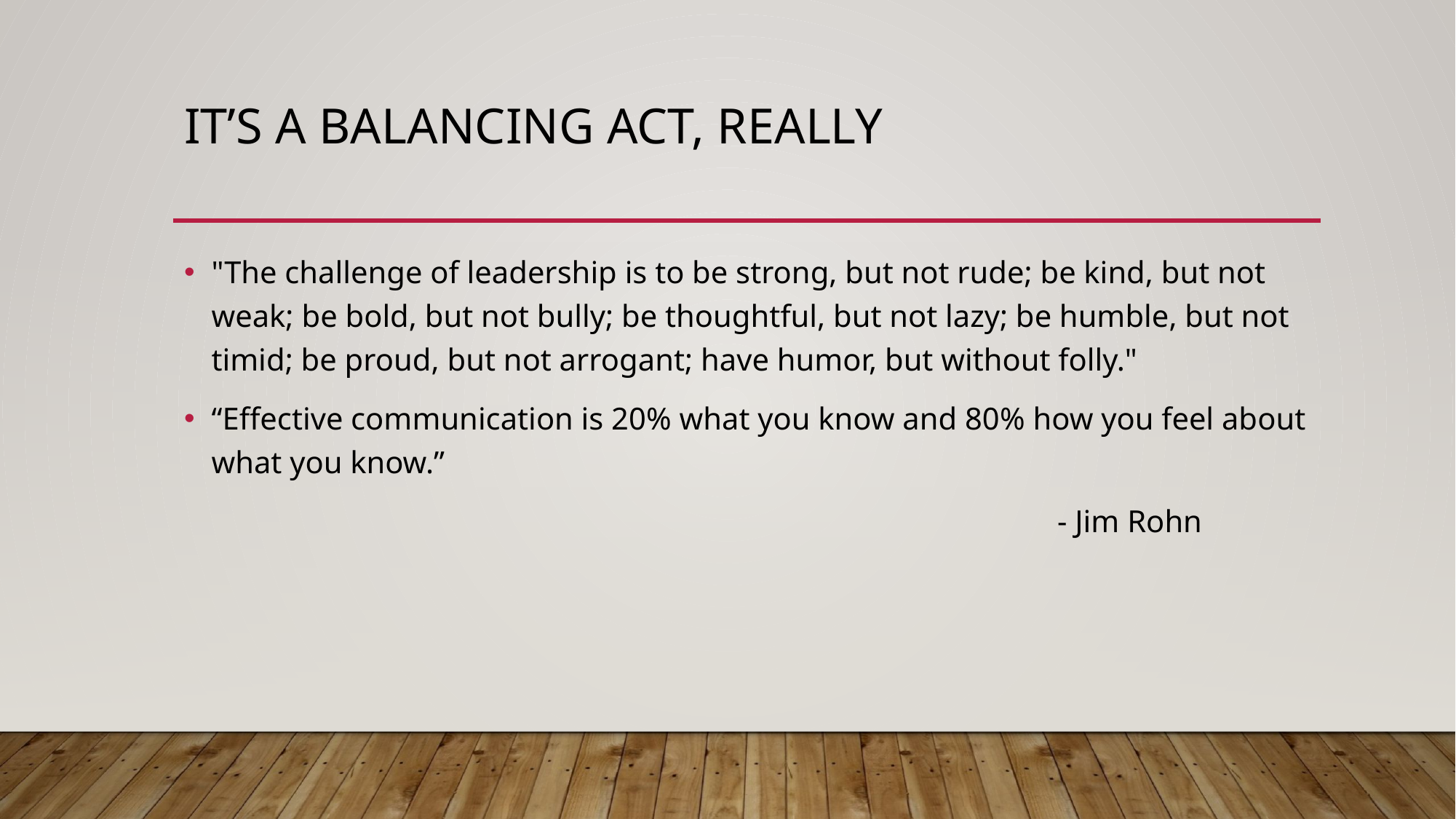

# It’s a balancing act, really
"The challenge of leadership is to be strong, but not rude; be kind, but not weak; be bold, but not bully; be thoughtful, but not lazy; be humble, but not timid; be proud, but not arrogant; have humor, but without folly."
“Effective communication is 20% what you know and 80% how you feel about what you know.”
								- Jim Rohn

## Slide 10
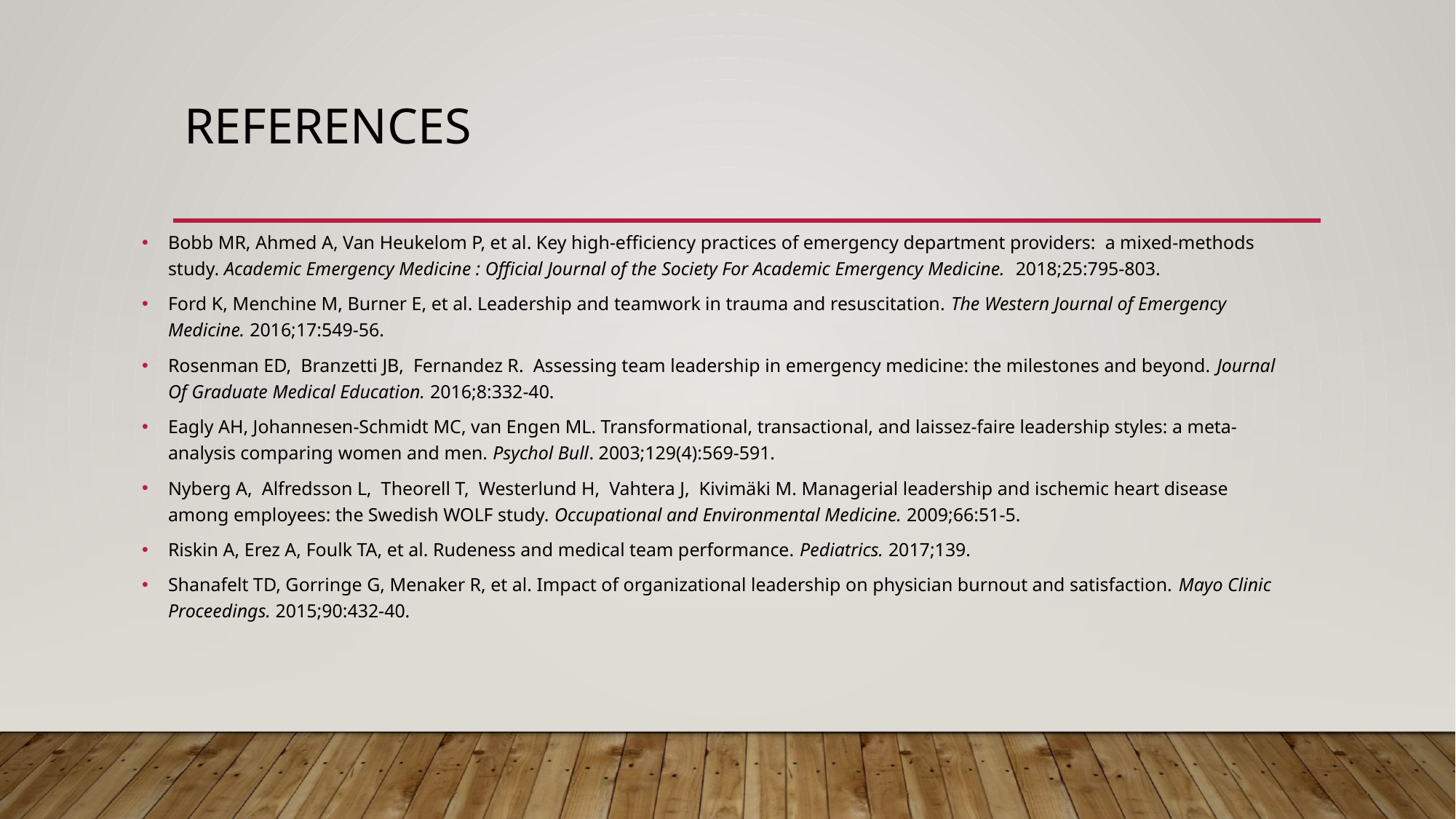

# References
Bobb MR, Ahmed A, Van Heukelom P, et al. Key high-efficiency practices of emergency department providers: a mixed-methods study. Academic Emergency Medicine : Official Journal of the Society For Academic Emergency Medicine. 2018;25:795-803.
Ford K, Menchine M, Burner E, et al. Leadership and teamwork in trauma and resuscitation. The Western Journal of Emergency Medicine. 2016;17:549-56.
Rosenman ED, Branzetti JB, Fernandez R. Assessing team leadership in emergency medicine: the milestones and beyond. Journal Of Graduate Medical Education. 2016;8:332-40.
Eagly AH, Johannesen-Schmidt MC, van Engen ML. Transformational, transactional, and laissez-faire leadership styles: a meta-analysis comparing women and men. Psychol Bull. 2003;129(4):569-591.
Nyberg A, Alfredsson L, Theorell T, Westerlund H, Vahtera J, Kivimäki M. Managerial leadership and ischemic heart disease among employees: the Swedish WOLF study. Occupational and Environmental Medicine. 2009;66:51-5.
Riskin A, Erez A, Foulk TA, et al. Rudeness and medical team performance. Pediatrics. 2017;139.
Shanafelt TD, Gorringe G, Menaker R, et al. Impact of organizational leadership on physician burnout and satisfaction. Mayo Clinic Proceedings. 2015;90:432-40.
